# Shared Human Leukocyte Antigen (HLA) Coverage in dementia and Parkinson’s disease

**DOI:** 10.29245/2572.942x/2020/3.1275

**Published:** 2020-09-22

**Authors:** Lisa M. James, Apostolos P. Georgopoulos

**Affiliations:** 1 Brain Sciences Center, Department of Veterans Affairs Health Care System, Minneapolis, MN, 55417, USA; 2 Department of Neuroscience, University of Minnesota Medical School, Minneapolis, MN 55455, USA; 3 Department of Psychiatry, University of Minnesota Medical School, Minneapolis, MN 55455, USA; 4 Department of Neurology, University of Minnesota Medical School, Minneapolis, MN 55455, USA

**Keywords:** Dementia, Parkinson’s Disease, Human Leukocyte Antigen, Immunity, Genetics

## Abstract

Dementia and Parkinson’s disease are the two most common age-related neurodegenerative conditions. Recent studies have identified Human Leukocyte Antigen (HLA) Class II DRB1 alleles that are protective or neutral with respect to dementia. Here we extend those findings to evaluate the association of the population frequency of HLA DRB1 alleles with the prevalence of dementia and Parkinson’s disease in14 Continental Western European countries. Nine HLA DRB1 alleles were identified including four that are protective against dementia (DRB1*01:01, DRB1*04:01, DRB1*13:02, DRB1*15:01), three that are neutral (DRB1*03:01, DRB1*07:01, DRB1*08:01), and two susceptibility alleles (DRB1*11:01, DRB1*04:05). Results demonstrated that the population prevalence’s of dementia and Parkinson’s disease are highly correlated and that the association between the nine DRB1 alleles above and the population prevalence of dementia is highly overlapping with that of Parkinson’s disease. These findings suggest a common HLA Class II DRB1 profile. Given the diverse role of HLA Class II alleles in protection from foreign antigens, autoimmunity, and, possibly, neuroprotection, the shared HLA profile between dementia and Parkinson’s disease indicates that common immunogenetic mechanisms underlie the pathogenesis and manifestation of these diseases.

## Introduction

Dementia and Parkinson’s disease are the most common age-related neurodegenerative conditions, together affecting an estimated 50 million people worldwide according to recent epidemiological studies^1,2^. Dementia refers to conditions reflecting a decline in cognitive, behavioral, motor, and/or social functioning; Alzheimer’s disease is the most common type, accounting for up to 80% of dementia cases^3^. AD is characterized primarily by impairments in memory and executive functioning although effects on mood, personality, and motor symptoms are also common. Parkinson’s disease is characterized primarily by motor symptoms including bradykinesia, rigidity, and tremor, yet non-motor symptoms including cognitive and mood disruptions are common, particularly as the disease progresses^4^. A large percentage of patients with Parkinson’s Disease eventually develop dementia^5^. In light of the overlap in symptoms and the substantial and increasing global burden of these conditions^1,2^, research aimed at identifying putative risk and protective factors are of paramount importance to mitigate the significant impacts of these condition on global health.

Although the etiology of dementia and Parkinson’s disease is unknown, various genetic and environmental risk and protective factors have been identified. For dementia, risk factors include possession of apolipoprotein E4 allele, health conditions such as cardiovascular disease and Type II diabetes, modifiable lifestyle factors such as poor diet and sedentary lifestyle, and exposure to infectious agents^6,7^. For Parkinson’s disease, variations in genes associated with alpha-synuclein and other proteins have been implicated^8^, and environmental risk factors include exposure to pollutants, pesticides, and infectious agents, dairy consumption, and β2- adrenoreceptor agonists (2,9,10,11). With regard to protection, adherence to plant-based diet as well as physical, social, and intellectual activities have been shown to protect against dementia^6^; environmental factors that protect against Parkinson’s disease include smoking, caffeine and tea intake, physical activity, vitamin E intake, gout, non-steroidal anti-inflammatory drugs and β2- adrenoreceptor agonists^10^. In terms of genetic protection, studies have demonstrated that apolipoprotein E2 protects against dementia^12,13^ and may protect against Parkinson’s disease-related dementia ^14,15^. Moving beyond apolipoprotein E, recent studies have also demonstrated robust genetic protection against dementia conferred by specific Class II human leukocyte antigen (HLA) alleles^16,17^. The extent to which those HLA alleles protect against Parkinson’s disease is unknown.

HLA genes code for cell-surface glycoproteins that are instrumental in immune response to foreign antigens such as viruses and bacteria. Class I HLA proteins present intracellular foreign antigen peptides to cytotoxic T cells to signal cell destruction. Class II HLA proteins present peptides to CD4 receptors to promote antibody production and elimination of foreign antigens. However, HLA genes, which are the most highly polymorphic in the human genome, differ in their ability to bind with foreign antigens and mount an immune response. In the absence of sufficient binding affinity and/or immunogenicity, antigenic peptides may persist. We have speculated that persistent antigens lead to neuronal damage and inflammation, and may contribute to dementia and other neuroimmune conditions^18–20^.

Using a publicly available repository^21^ containing HLA data pooled from histocompatibility laboratories and published studies, coupled with dementia prevalence obtained from the Global Burden of Disease studies^1^ we have examined associations between HLA and dementia prevalence in 14 countries in Continental Western Europe where dementia prevalence is among the highest in the world. We demonstrated that three specific Class II HLA DRB1 alleles – DRB1*01:01, DRB1*13:02, and DRB1*15:01 - are highly protective against dementia, even after accounting for apolipoprotein E4^17^. Moreover, the combined population frequency of the three alleles accounted for 67% of the variance in dementia prevalence and 48% of the change in dementia prevalence in those countries over the last 25 years. Given the role of Class II HLA in antibody production and host protection against re-exposure to foreign antigens, the finding that dementia prevalence is lower in countries with higher frequency of these alleles suggests that lower dementia rates may be partially attributable to protection against pathogens that bind with these alleles. In fact, a subsequent in silico study revealed that these three dementia alleles were found to bind with significantly higher affinity to herpes virus antigens than were three HLA alleles that are dementia-neutral - DRB1*03:01, DRB1*07:01, DRB1*08:01^22^. In the present study, we extend those findings to investigate the association of the population frequency^21^ of HLA -DRB1 dementia-protective, dementia-neutral alleles to Parkinson’s disease population prevalence^2^.

## Methods

### CWE countries.

As in our previous studies^16,17^ we focused on the following 14 CWE countries: Austria, Belgium, Denmark, Finland, France, Germany, Greece, Italy, Netherlands, Portugal, Norway, Spain, Sweden, and Switzerland.

### Prevalence of dementia and Parkinson’s disease.

The Global Burden of Disease study is the most comprehensive epidemiological study of worldwide morbidity and mortality associated with several conditions including dementia^1^ and Parkinson’s disease^2^. The total number of people with dementia or Parkinson’s disease in each of the 14 CWE countries was retained for analyses in the present study. The Population Reference Bureau^23^ provides vital demographic data for over 200 countries based on several sources including official national data or analyses conducted by national, regional, or worldwide (e.g. United Nations Population Division) offices. The prevalences of dementia and Parkinson’s disease were computed by dividing the number of people with dementia^1^ or Parkinson’s disease^2^ in 2016 in each country by the total population of the country in 2016^23^ and expressed as a percentage. These data are shown in Table 1. We have previously shown that life expectancy for these countries are virtually identical^3^ (Table 1); therefore, life expectancy was not included in the current analyses.

### HLA alleles.

In our previous studies we identified three HLA Class II alleles protective for dementia^17^ (DRB1*01:01, DRB1*13:02, DRB1*15:01) and three alleles that are neutral for dementia^21^ (DRB1*03:01, DRB1*07:01, DRB1*08:01); in addition, we now identified an allele protective for dementia (DRB1*04:01; Allele Frequency Net Database, http://www.allelefrequencies.net^21^). To obtain allele frequencies, the Allele Frequency Net Database was queried to provide the 4-digit HLA allele frequency for each country with no further restrictions by region, ethnicity, source of study, or other qualifiers. For countries in which were available for more than one population we obtained the average frequency. The frequencies of these seven alleles for each country are given in Table 2.

### Statistical analyses.

Regression and correlation analyses were used to evaluate the association between the population frequency of each the seven HLA alleles above (Table 2), alone and combined, and the prevalence of dementia and Parkinson’s disease in Continental Western Europe. Previous analyses^16,17^ had documented an exponential relation between dementia prevalence and allele frequency; hence, the prevalences of dementia and Parkinson’s disease were natural-log transformed to analyze their relation to allele frequency using linear regression. Pairwise Pearson correlation coefficients were compared by calculating the normal deviate z-statistic^24^. Regression coefficients were compared, as needed, using the following formula^25^:

(1) $$z=\frac{b_{1}-b_{2}}{\sqrt{{SEb_{1}}^{2}+{SEb_{2}}^{2}}}$$

where Z is the normal deviate, $b_{1}$ and $b_{2}$ denote regression coefficients to be compared, and *SE* are their standard errors. Finally, correlation coefficients r were Fisher z-transformed^24^ $r^{\prime }$ to normalize their distribution:

(2) $$r^{\prime }=\text{atanh}(r)$$

All statistical analyses were conducted using the IBM-SPSS statistical package (version 23).

## Results

Association of dementia and Parkinson’s disease prevalences

**Figure 1.**
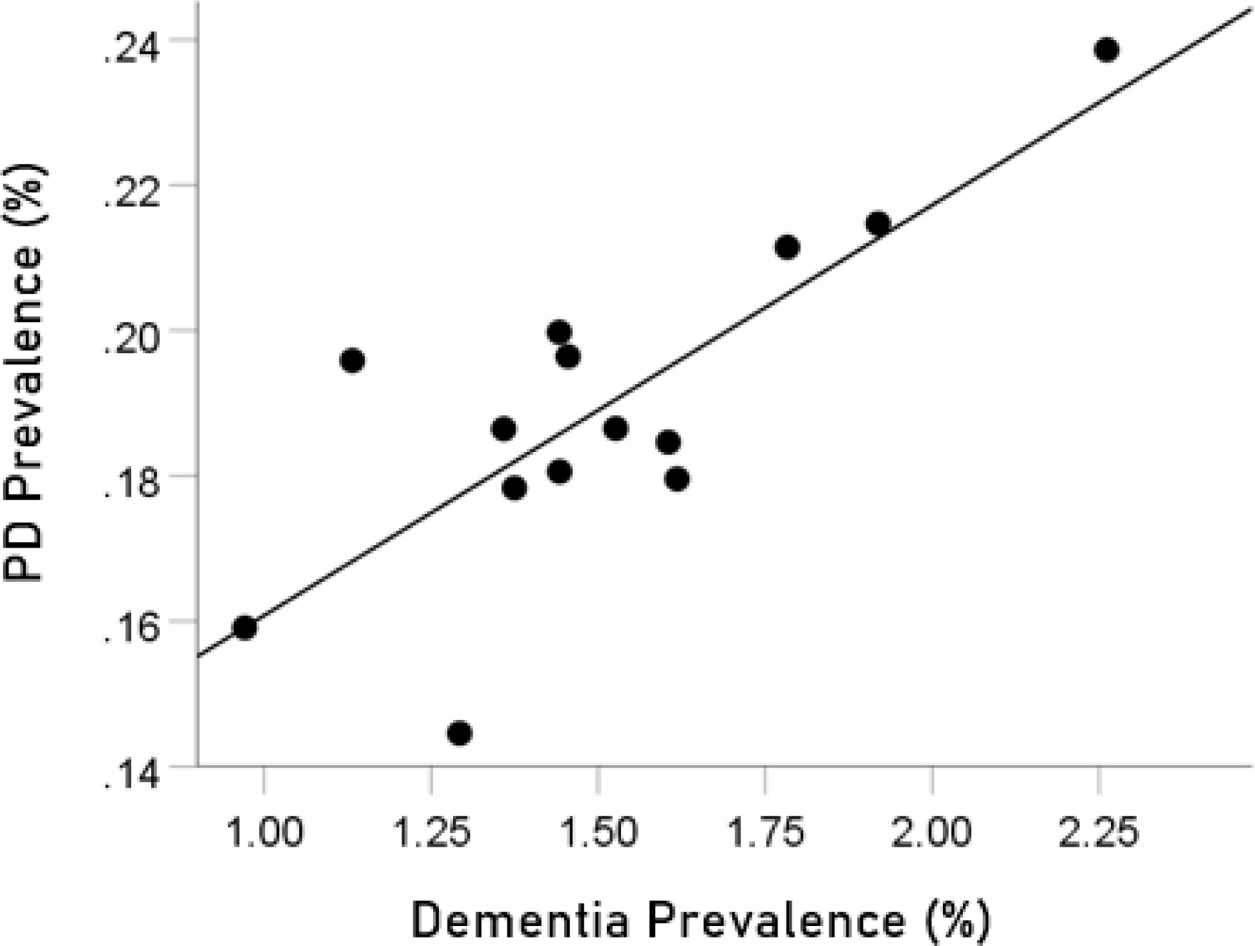
Dementia prevalence in 14 CWE countries is plotted against the prevalence of Parkinson’s disease. See text for details.

The prevalences of dementia and Parkinson’s disease were highly significantly correlated (Figure 1; r=0.787, P=0.000843, N=14).

### Protective HLA alleles

#### Association between dementia and Parkinson’s disease prevalences and the frequencies of four protective HLA alleles

**Figure 2.**
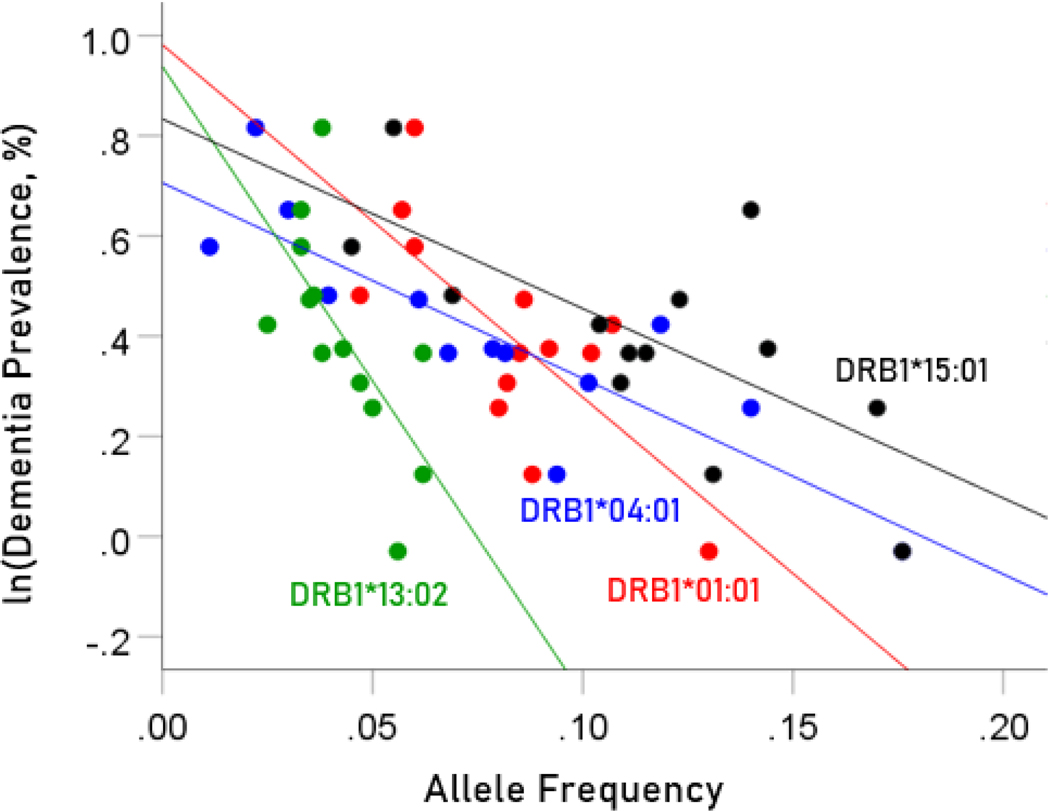
Illustration of the different effects of each protective allele on the natural log of dementia prevalence as evidenced by the varying steepness of the slopes.

**Figure 3.**
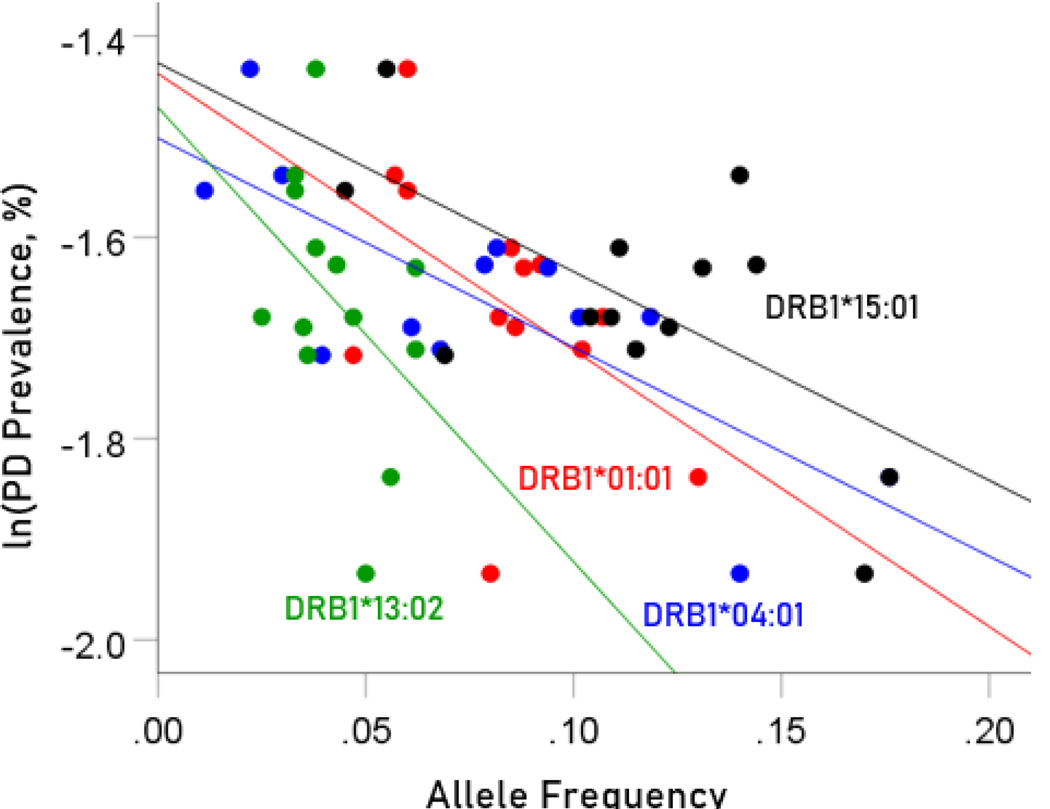
Illustration of the different effects of each protective allele on the natural log of Parkinson’s disease prevalence as evidenced by the varying steepness of the slopes.

The values of log-transformed dementia and Parkinson’s disease prevalences are plotted against the frequencies of the four protective (negatively associated) alleles in Figure. 2 and 3, respectively. The corresponding correlation coefficients are given in Table 3. There were no statistically different differences between any of the dementia- and Parkinson’s disease correlations (Table 3).

#### Association of dementia and Parkinson’s disease prevalences with the combined frequencies of the four protective alleles

**Figure 4.**
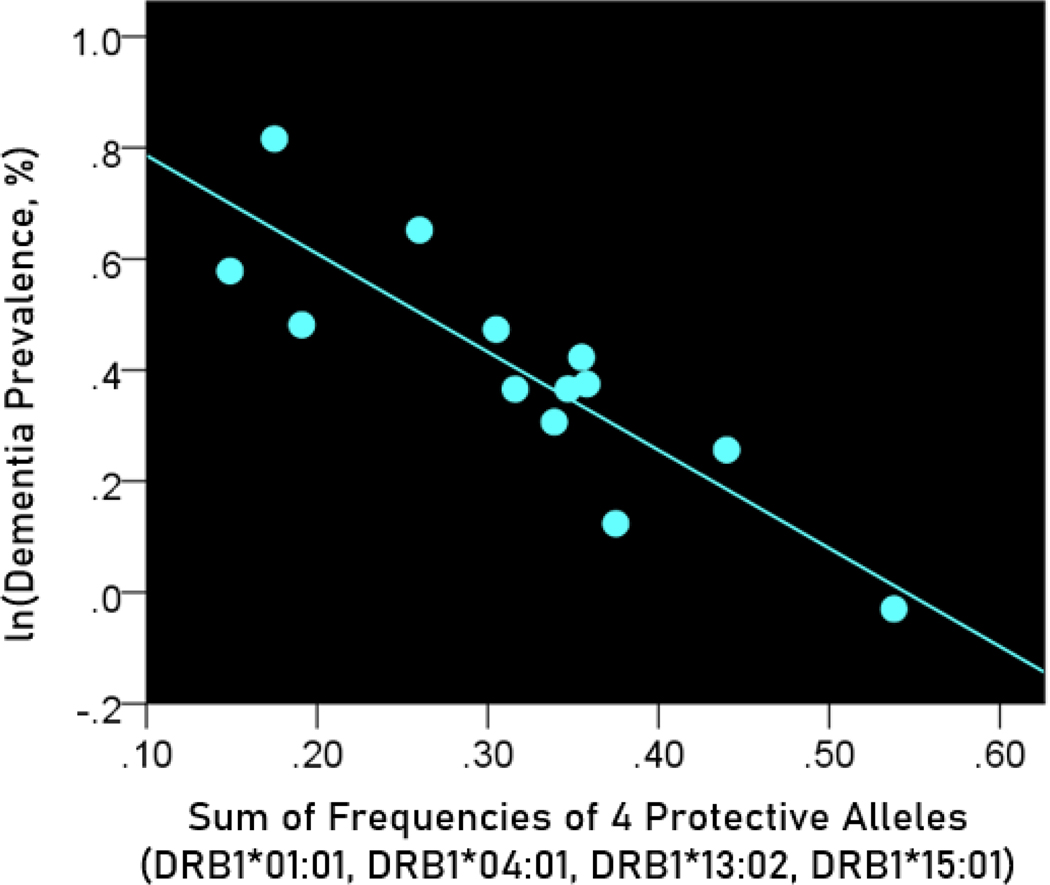
The natural log of dementia prevalence is plotted against the sum of the frequencies of the four protective alleles (DRB1*01:01, DRB1*04:01, DRB1*13:02, DRB1*15:01). See text for details.

The log-transformed prevalence of dementia is plotted against the sum of the frequencies of four protective alleles in Figure 4:

(3) $$f_{\left[4\;\mathrm{protective alleles}\right]}=f_{DRB1*01:01}+f_{DRB1*04:01}+f_{DRB1*13:02}+f_{DRB1*15:01}$$

The regression equation was as follows

(4) ln(dementia prevalence, %)=0.963−1.766(± 0.301)f[4protective alleles]+error

${\text{r}}^{2}=0.758$, P=0.001; value in parenthesis is the SE of the regression coefficient.

**Figure 5.**
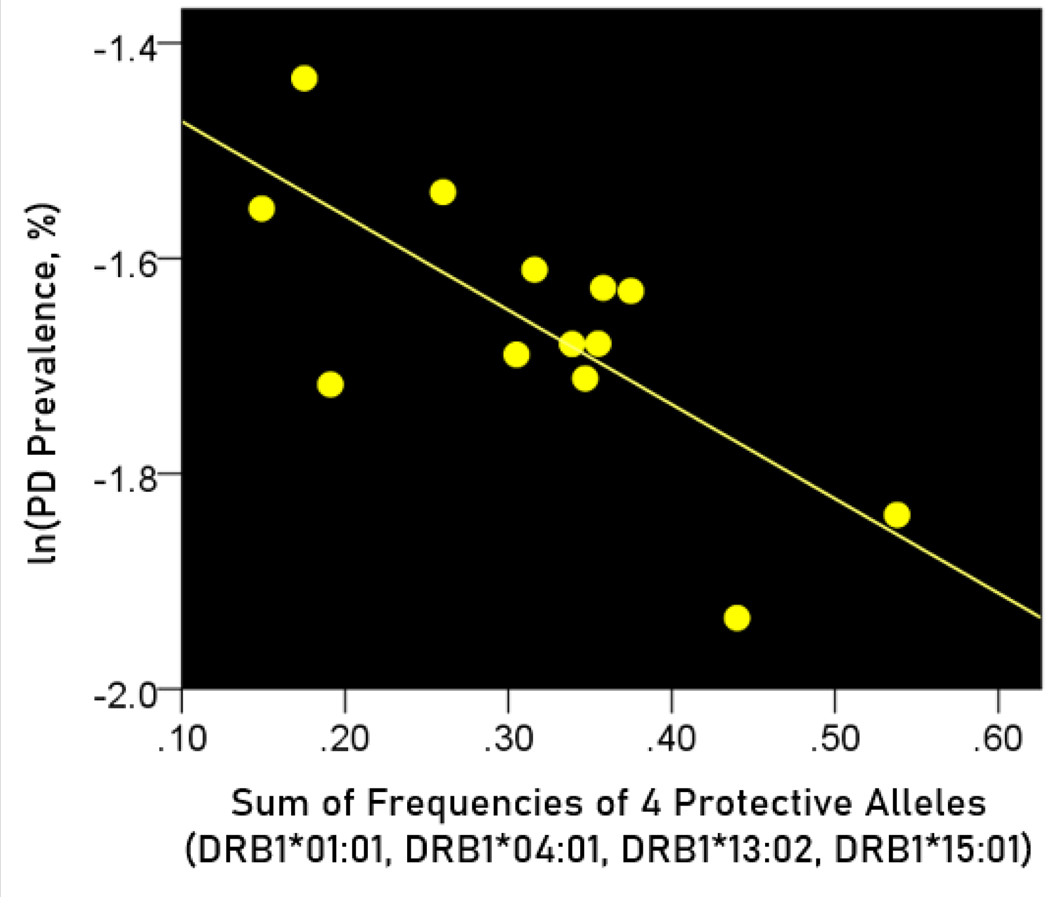
The natural log of Parkinson’s disease prevalence is plotted against the sum of the frequencies of the four protective alleles (DRB1*01:01, DRB1*04:01, DRB1*13:02, DRB1*15:01). See text for details.

The log-transformed prevalence of Parkinson’s disease (PD) is plotted against the sum of the frequencies four protective alleles in Figure 5. The regression equation was as follows.


(5) ln(PD prevalence, %)=−1.385−0.876(± 0.241)f[4protective alleles]+error

${\text{r}}^{2}=0.546$, P=0.004; value in parenthesis is the SE of the regression coefficient.

The f[4protective alleles] regression coefficient for dementia (−1.766, equation 3) was 2x more negative (i.e. twice as protective) for dementia than that for Parkinson’s disease (-0.876, equation 4), a highly significant difference (z=5.36, P<0.001, test of equation 1).

### Neutral alleles

#### Association between dementia and Parkinson’s disease prevalences and the frequencies of three neutral HLA alleles

**Figure 6.**
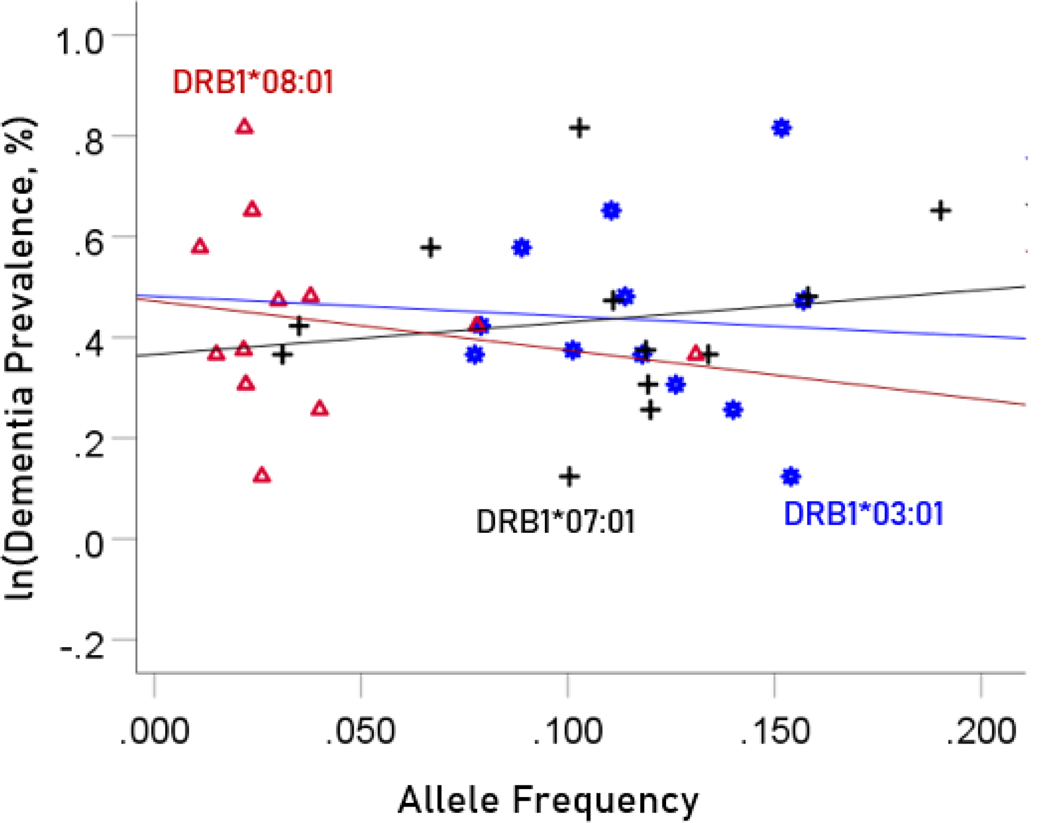
The natural log of dementia prevalence is plotted against the frequency of the three neutral alleles; there is no significant association. See text for details.

**Figure 7.**
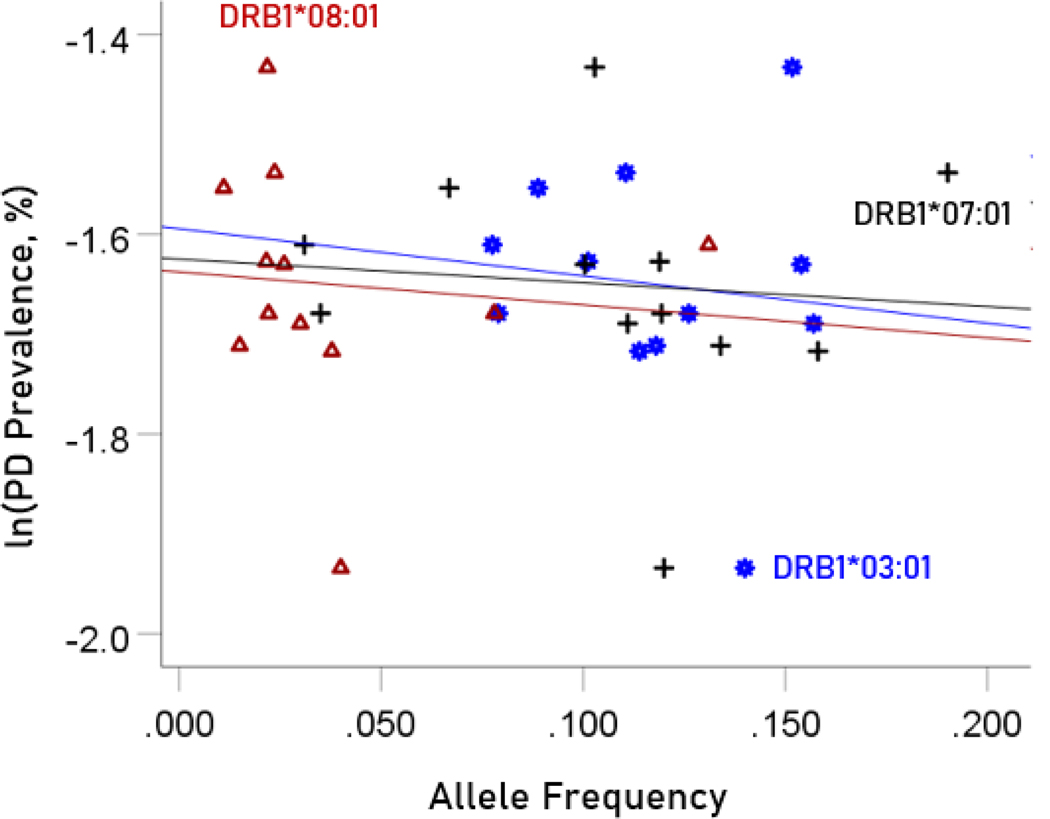
The natural log of Parkinson’s disease prevalence is plotted against the frequency of the three neutral alleles; there is no significant association. See text for details.

The values of log-transformed dementia and Parkinson’s disease prevalences are plotted against the frequencies of the three neutral (not significantly associated) alleles in Figure. 6 and 7, respectively. The corresponding correlation coefficients are given in Table 3. There were no statistically different differences between any of the dementia- and Parkinson’s disease correlations (Table 3).

#### Association of dementia and Parkinson’s disease prevalences with the combined frequencies of the three neutral alleles

**Figure 8.**
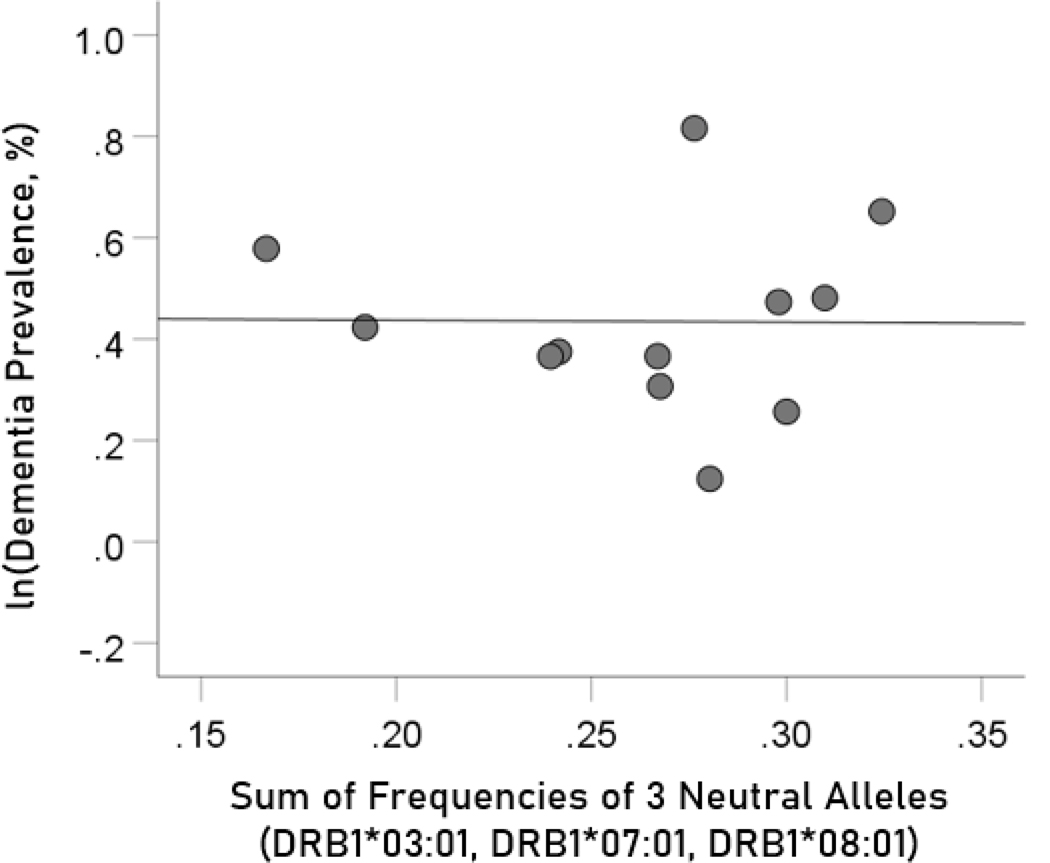
The natural log of dementia prevalence is plotted against the sum of the frequencies of the three neutral (DRB1*03:01, DRB1*07:01, DRB1*08:01); there is no significant association. See text for details.

The log-transformed prevalence of dementia is plotted against the sum of the frequencies of the three neutral alleles in Figure 8:

(6) $$f_{\left[3neutra\mathrm{l a}lleles\right]}=f_{DRB1*03:01}+f_{DRB1*07:01}+f_{DRB1*08:01}$$

The regression equation was as follows.


(7) ln(dementia prevalence, %)=0.446−0.41(± 1.239)f[3neutral alleles]+error

${\text{r}}^{2}=0.0001$, P=0.974; value in parenthesis is the SE of the regression coefficient. There was no statistically significant association.

**Figure 9.**
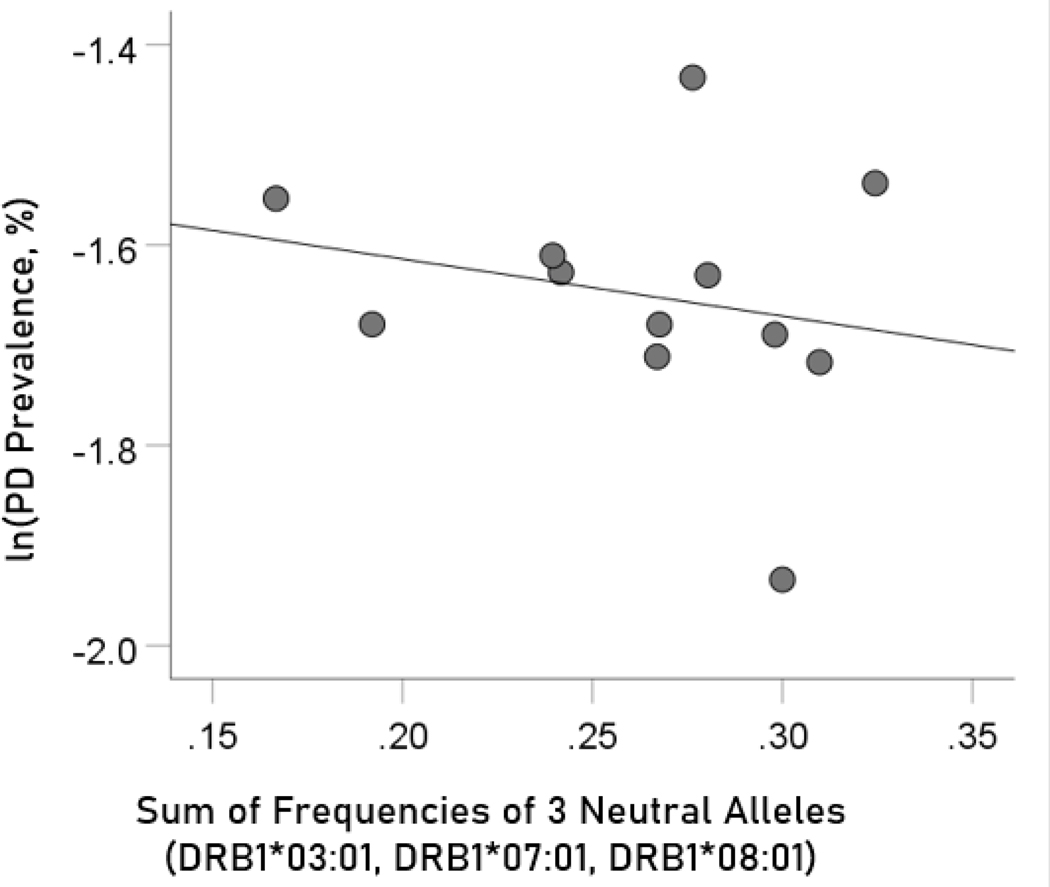
The natural log of Parkinson’s disease prevalence is plotted against the sum of the frequencies of the three neutral (DRB1*03:01, DRB1*07:01, DRB1*08:01); there is no significant association. See text for details.

The log-transformed prevalence of Parkinson’s disease is plotted against the sum of the frequencies four protective alleles in Figure 9. The regression equation was as follows.


(8) ln(Parkinson's disease prevalence, %)=−1.5−0.571(± 0.799)f[3neutral alleles]+error

r^2^ = 0.049, P = 0.491; value in parenthesis is the SE of the regression coefficient. There was no statistically significant association.

### Predisposing HLA alleles

#### Association between dementia and Parkinson’s disease prevalences and the frequencies of two predisposing HLA alleles

**Figure 10.**
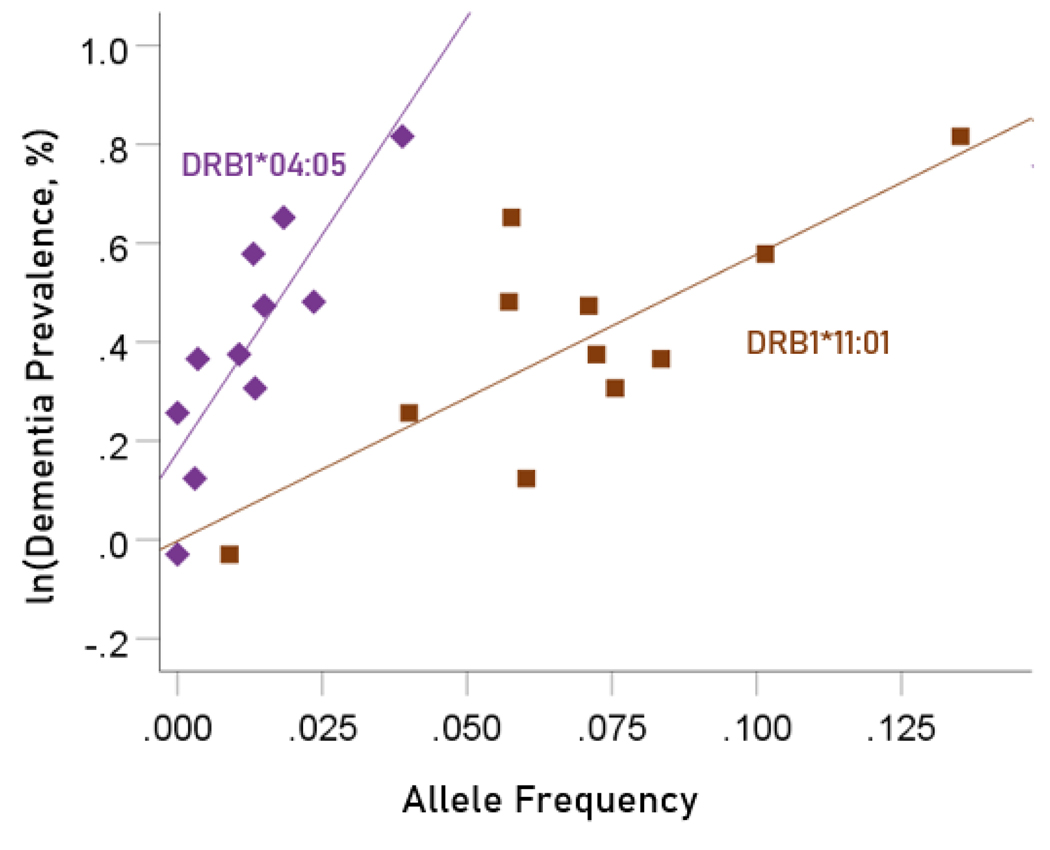
Illustration of the different effects of each predisposing allele on the natural log of dementia prevalence as evidenced by the varying steepness of the slopes.

**Figure 11.**
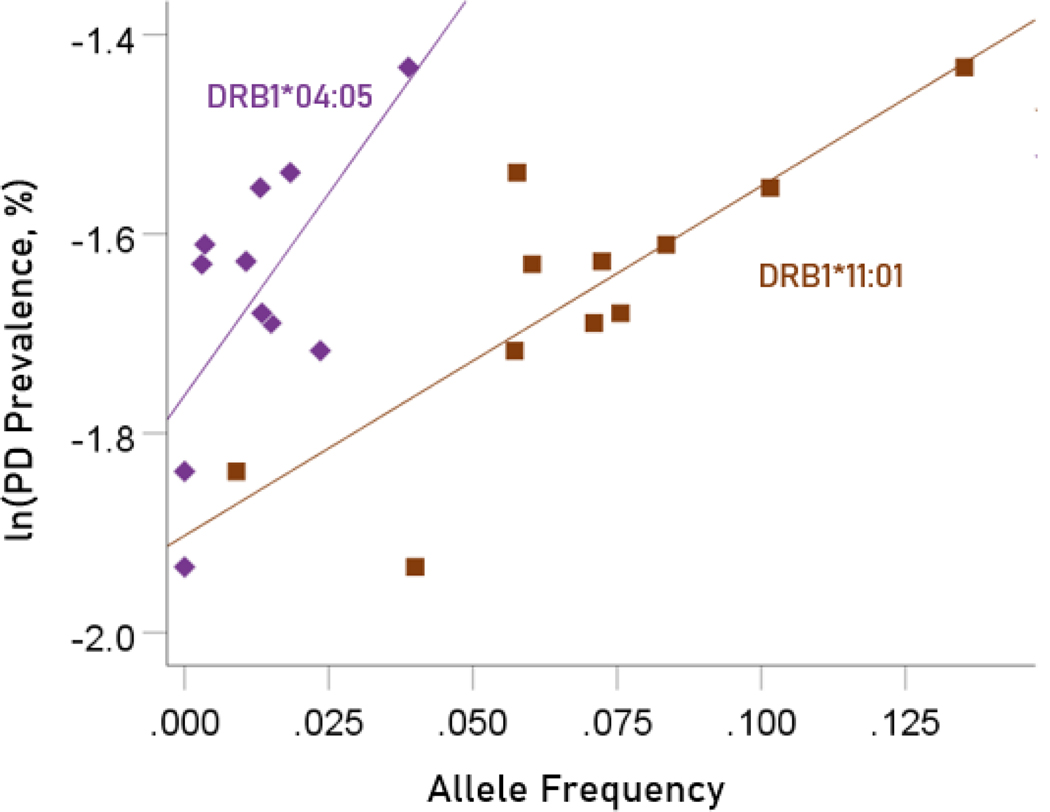
Illustration of the different effects of each predisposing allele on the natural log of Parkinson’s disease prevalence as evidenced by the varying steepness of the slopes.

The values of log-transformed dementia and Parkinson’s disease prevalences are plotted against the frequencies of the two predisposing alleles in Figure. 10 and 11, respectively. The corresponding correlation coefficients are given in Table 3. There were no statistically different differences between any of the dementia- and Parkinson’s disease correlations (Table 3).

#### Association of dementia and Parkinson’s disease prevalences with the combined frequencies of the three neutral alleles

**Figure 12.**
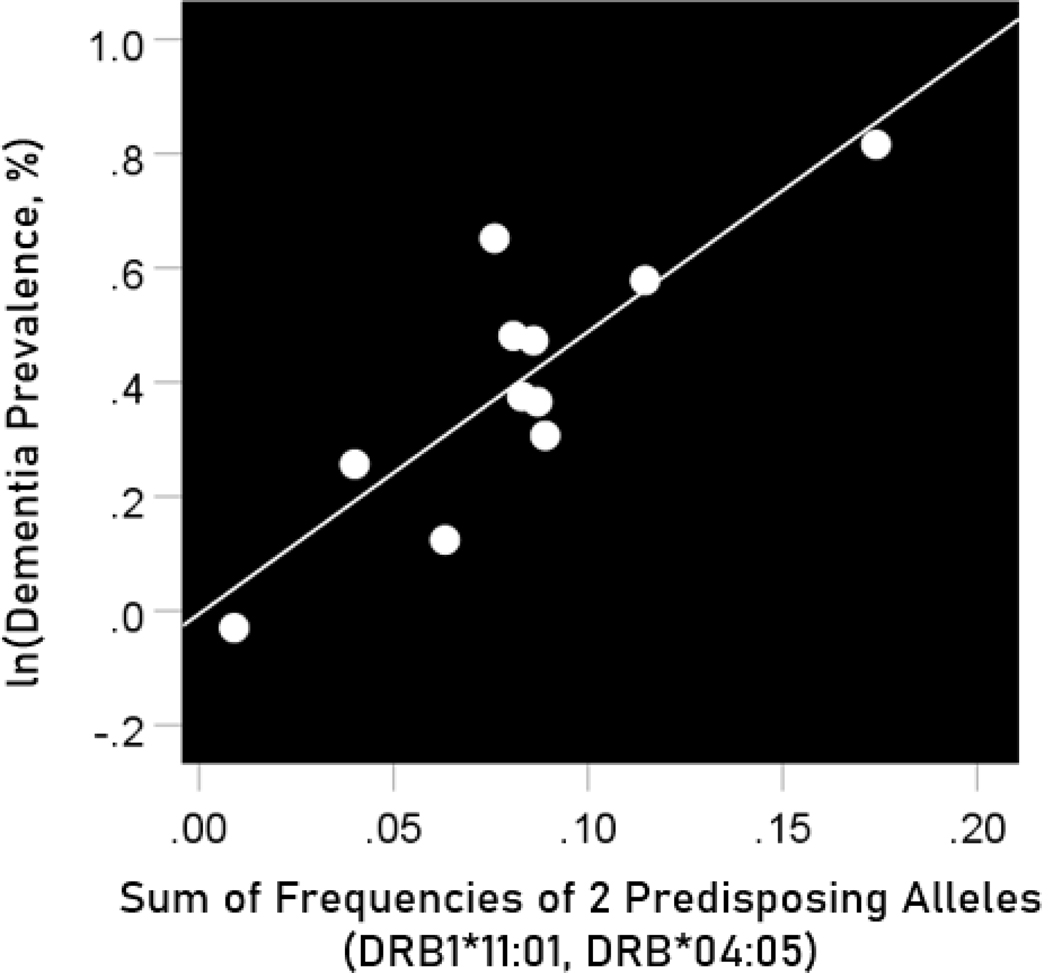
The natural log of dementia prevalence is plotted against the sum of the frequencies of the two predisposing alleles (DRB1*04:05, DRB1*11:01). See text for details.

The log-transformed prevalence of dementia is plotted against the sum of the frequencies of the three neutral alleles in Figure 12:

(9) $$f[2predisposin\mathrm{g a}lleles]=f_{DRB1*04:05}+f_{DRB1*11:01}$$

The regression equation was as follows.


(10) ln(dementia prevalence, %)=−0.005+4.939(± 1.002)f[2predisposing alleles]+error

${\text{r}}^{2}=0.729$, P=0.001; value in parenthesis is the SE of the regression coefficient.

**Figure 13.**
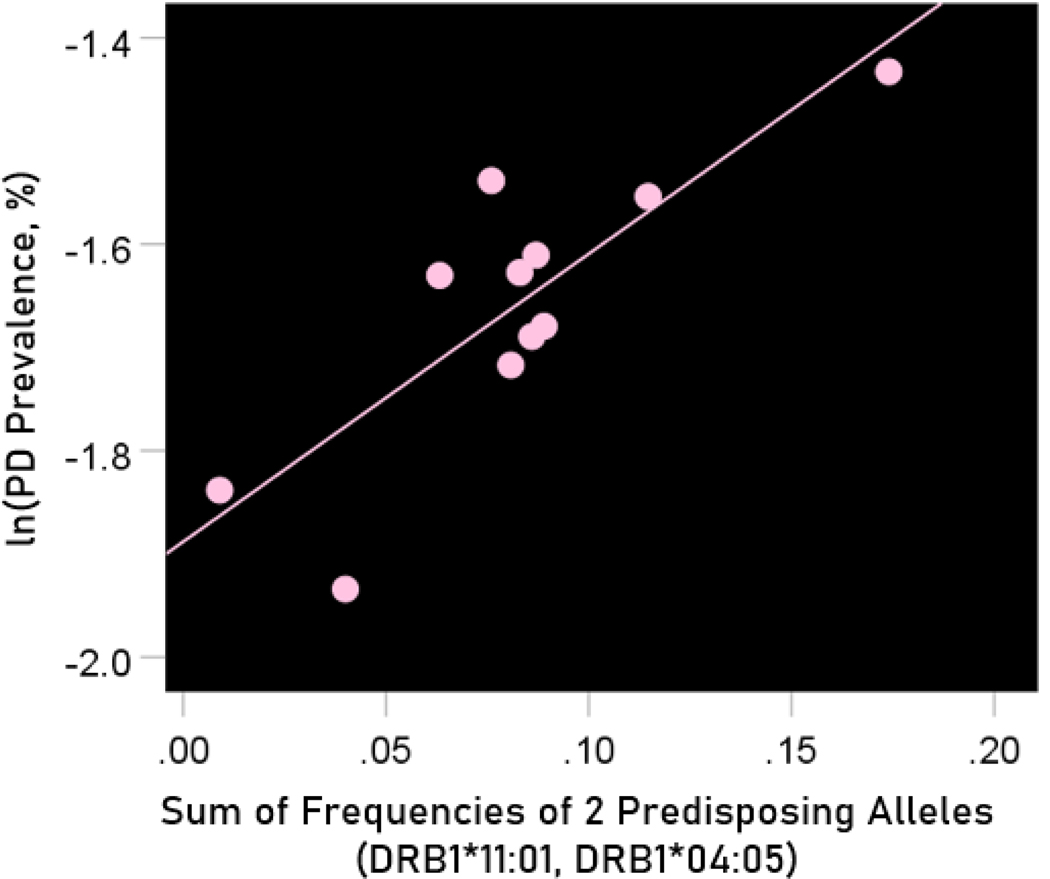
The natural log of Parkinson’s disease prevalence is plotted against the sum of the frequencies of the two predisposing alleles (DRB1*04:05, DRB1*11:01). See text for details.

The log-transformed prevalence of Parkinson’s disease is plotted against the sum of the frequencies four protective alleles in Figure 13. The regression equation was as follows.


(11) ln(Parkinson's disease prevalence, %)=−1.888+2.789(± 0.626)f[3neutral alleles]+error

${\text{r}}^{2}=0.688$, P=0.002; value in parenthesis is the SE of the regression coefficient.

### HLA profile of a disease

A given disease may have varying associations with different HLA alleles. In the context of populations, this relation would be captured by the correlation between disease prevalence and allele frequency, since both of these measures typically vary across population samples (e.g. countries, ethnicities, etc.): this variation provides the requisite variety that makes disease-HLA associations possible. Let $p_{i}^{k=1,M}$ be the prevalence of disease i in a sample population K (out of M available); let $f_{j=1,N}^{k=1,M}$ be the frequency of a HLA allele j (out of N available) in that population. We call the vector of z-transformed correlations $r^{\prime }$ between the prevalence of the disease i in M populations and the frequency of N alleles in the same populations the “HLA profile of disease $i^{\prime \prime }$. In the present study, the HLA profiles of dementia and Parkinson’s disease, for the 9 HLA alleles studied, are shown in Table 4. Specifically, M=14 CWE countries, N=9 HLA alleles.

#### Association between HLA disease profiles

**Figure 14.**
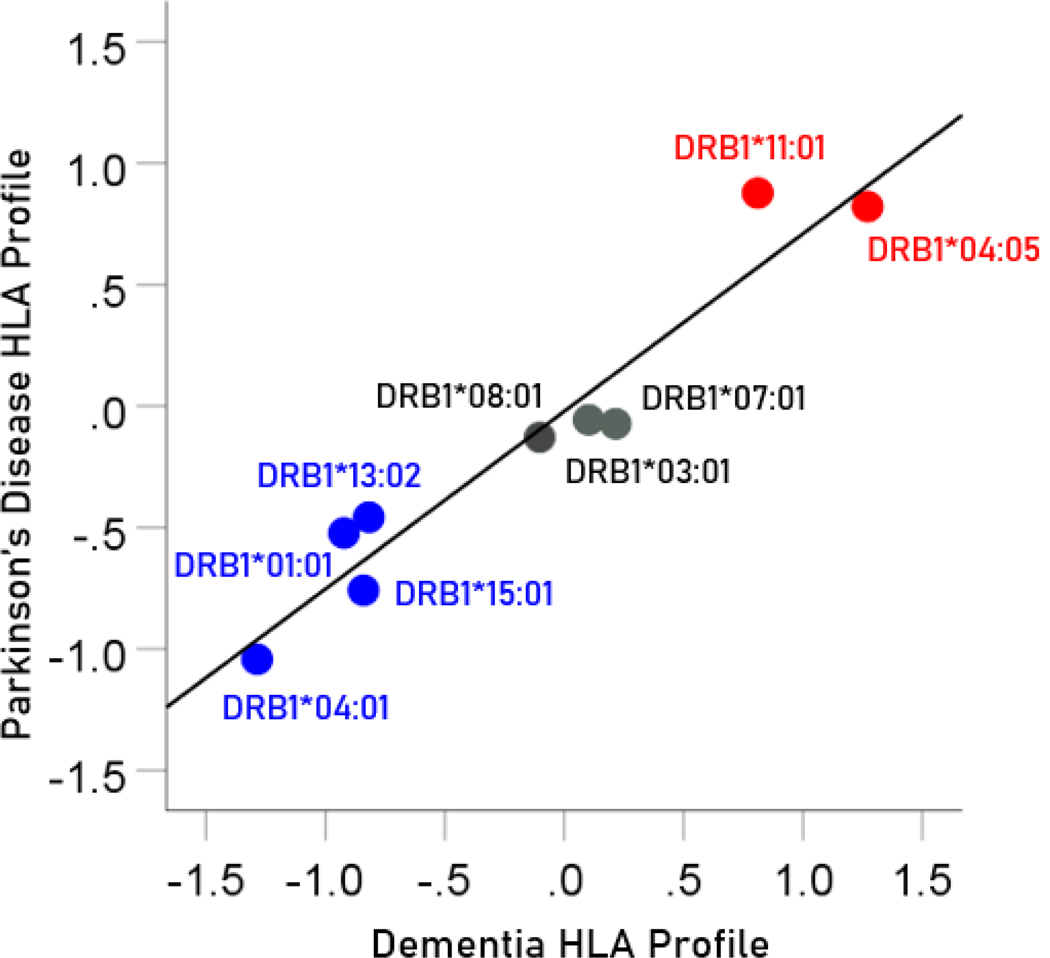
The HLA profile of Parkinson’s disease is plotted against the HLA profile of dementia; data from Table 4. Blue, protective alleles; black, neutral alleles; red, predisposing alleles.

The pairwise association between HLA disease profiles provides a quantitative estimate of the similarity of dependence of disease prevalence to an ensemble of HLA alleles. Since HLA alleles can be involved in pathogen elimination (positive aspect) and/or autoimmunity (negative aspect), determining the similarity between HLA disease profiles would provide an overall assessment of the similarity of involvement of such factors in the specific diseases in a pair. Since the HLA profile consists of z-transformed correlations, the Pearson correlation between two HLA disease profiles would give a quantitative assessment of the association, including its statistical significance and the percent of HLA profile variance explained. In this study, there was a highly positive association between the dementia and Parkinson’s HLA disease profiles (Figure 14); the correlation coefficient was 0.965 (P=0.000026; $r^{2}=0.931$) and the percent of variance explained $r^{2}\times 100=93.1\text{\%}$.

## Discussion

Here we investigated the association of the population frequency of seven HLA Class II DRB1 alleles with respect to the prevalence of dementia and Parkinson’s disease in 14 countries in Continental Western Europe. Several findings emerged. First, the population prevalence of dementia and Parkinson’s disease were positively and highly significantly associated. Second, the average frequency of protective HLA alleles accounted for substantial variance in the population prevalence of dementia and, to a lesser extent, Parkinson’s disease. Finally, the associations of disease prevalence and the HLA alleles were highly similar for dementia and Parkinson’s disease. Taken together, these findings point to a common HLA profile, suggesting shared immunogenetic mechanisms (protective, neutral, and predisposing) underlying these diseases.

HLA Class II alleles, to which the DRB1 alleles investigated here belong, play a critical role in facilitating antibody production and immunological memory to foreign antigens. In light of that role, the highly protective effects of the four DRB1 alleles identified here can be interpreted as likely promoting pathogen elimination that may otherwise contribute to disease susceptibility. That is, those lacking immunogenetic protection conferred by possession of one or more of these DRB1 alleles (or other HLA alleles with similar sequences and, consequently, similar binding affinity) may be unable to sufficiently eliminate certain pathogens, leading to deleterious downstream effects that may include dementia and/or Parkinson’s disease. Indeed, several viral and bacterial pathogens have been implicated in both dementia^7^ and Parkinson’s disease^9,11^, many of which overlap across conditions consistent with the highly overlapping HLA profile observed here. In particular, human herpes viruses have been implicated in both conditions^26–29^, which is notable in light of a recent in silico study that demonstrated superior binding affinity of three of the HLA studies investigated here to human herpes viruses when compared to dementia-neutral HLA alleles^22^. Additional studies are currently underway to identify other antigens that bind with these protective HLA alleles and that, conversely, may be associated with dementia and/or Parkinson’s disease in the absence of HLA protection. Finally, with respect to predisposing HLA Class II alleles, such as those identified in this study, it is very likely that they are involved in autoimmunity mechanisms in both Alzheimer’s and Parkinson disease, as discussed recently^30^.

Although the current findings identified protective effects of four HLA DRB1 alleles, they neither preclude the possibility that other Class I or Class II HLA alleles may confer similar protection or predisposition nor that the HLA alleles shown to protect against dementia and Parkinson’s disease may promote risk for other conditions. For instance, DRB1*04:01, one of the alleles shown to protect against dementia and Parkinson’s disease in the current study, is associated with elevated risk of Type I diabetes^31^ and rheumatoid arthritis^32^. Similarly, DRB1*15:01 is a well-established risk factor for multiple sclerosis^33^, yet is protective against dementia and Parkinson’s disease at the population level. Research in our laboratory is underway to examine associations of other Class I and Class II HLA alleles with dementia, Parkinson’s disease, and other related conditions.

## Figures and Tables

**Table 1.** Dementia prevalence for Countries in Continental Western Europe. Overall Dementia Prevalence = (N Disease x 100) / (N total population)

Country	N Dementia^a^	N Parkinson’s Disease^b^	N total population^c^	Dementia Prevalence (%)	Parkinson’s Disease Prevalence (%)	Average life expectancy^c^
Austria	126914	15891	8800000	1.442	0.1806	81.5
Belgium	181350	20862	11300000	1.605	0.1846	81.5
Denmark	55336	9068	5700000	0.971	0.1591	80.5
Finland	83950	10258	5500000	1.526	0.1865	81.5
France	877760	120455	64600000	1.359	0.1865	82.0
Germany	1201668	162246	82600000	1.455	0.1964	80.5
Greece	192563	22837	10800000	1.783	0.2115	81.0
Italy	1370308	144606	60600000	2.261	0.2386	82.5
Netherlands	192425	33297	17000000	1.132	0.1959	81.5
Norway	67207	7517	5200000	1.292	0.1446	82.0
Portugal	166660	18496	10300000	1.618	0.1796	80.0
Spain	830915	92971	43300000	1.919	0.2147	82.5
Sweden	142735	19776	9900000	1.442	0.1998	82.0
Switzerland	115476	14979	8400000	1.375	0.1783	83.0

a Data obtained from ref^1^.

b Data obtained from ref^2^.

c Data obtained from ref^pop’n bureau^.

**Table 2.** Frequencies of 9 HLA Class II alleles for countries in Continental Western Europe used in this study.^a^ N/A, not available.

Country	DRB1*01:01	DRB1*03:01	DRB1*04:01	DRB1*04:05	DRB1*07:01	DRB1*08:01	DRB1*11:01	DRB1*13:02	DRB1*15:01
Austria	0.102	.118	0.068	N/A	.134	.015	.090	0.062	0.115
Belgium	0.086	.157	0.061	.015	.111	.030	.071	0.035	0.123
Denmark	0.130	.102	0.176	.000	N/A	.028	.009	0.056	0.176
Finland	0.107	.079	0.119	N/A	.035	.078	.023	0.025	0.104
France	0.082	.120	0.101	.013	.114	.024	.076	0.047	0.109
Germany	0.092	.103	0.079	.011	.114	.027	.072	0.043	0.144
Greece	0.060	.085	0.011	.013	.076	.011	.101	0.033	0.045
Italy	0.060	.152	0.022	.039	.103	.022	.135	0.038	0.055
Netherlands	0.088	.139	0.094	.003	.094	.023	.060	0.062	0.131
Norway	0.080	.140	0.140	.000	.120	.040	.040	0.050	0.170
Portugal	0.047	.114	0.039	.024	.158	.038	.057	0.036	0.069
Spain	0.057	.109	0.030	.018	.192	.024	.058	0.033	0.140
Sweden	0.085	.078	0.082	.004	.031	.131	.083	0.038	0.111
Switzerland	0.078	N/A	N/A	N/A	N/A	N/A	.076	0.053	0.109

a. Obtained from allelefrequencies.net October 19, 2019.

**Table 3.** Correlation statistics between disease prevalences and allele frequencies analyzed. P-values are in parentheses. Blue, protective alleles; black, neutral; red, predisposing.

	Alleles	Correlation Coefficients		Comparison of Correlations		
		ln(Dementia prevalence)	ln(Parkinson’s disease prevalence)			
		HLA Profile for Dementia	HLA Profile for Parkinson’s	Test statistic z	P value	N
1	DRB1*01:01	−0.727 (P=0.003)	−0.480 (0.082)	0.937	0.174	14
2	DRB1*04:01	−0.857 (0.00018)	−0.778 (0.002)	0.570	0.284	13
3	DRB1*13:02	−0.674 (0.008)	−0.428 (0.127)	0.846	0.199	14
4	DRB1*15:01	−0.686 (0.007)	−0.640 (0.014)	0.193	0.424	14
5	DRB1*03:01	0.102 (0.739)	−0.057 (0.854)	0.356	0.361	13
6	DRB1*07:01	0.212 (0.509)	−0.073 (0.821)	0.612	0.270	12
7	DRB1*08:01	−0.086 (0.780)	−0.046 (0.882)	0.090	0.464	13
8	DRB1*11:01	0.670 (0.009)	0.705 (0.005)	0.156	0.438	14
9	DRB1*04:05	0.854 (0.001)	0.676 (0.022)	0.898	0.185	11

**Table 4.** HLA profiles for dementia and Parkinson’s disease. Numbers are Fisher z-transformed correlation coefficients $r^{\prime }$. (See text for details.)

	HLA Profile for Dementia	HLA Profile for Parkinson’s
DRB1*01:01	−.0922	−0.523
DRB1*03:01	−1.286	−1.043
DRB1*04:01	−0.818	−0.457
DRB1*07:01	−0.840	−0.758
DRB1*08:01	0.102	−0.057
DRB1*13:02	0.215	−0.073
DRB1*15:01	−0.102	−0.129
DRB1*11:01	0.877	0.811
DRB1*04:05	0.822	1.271
